# Supplementary material for: Identification of Structural and Molecular Signatures Mediating Adaptive Changes in the Mouse Kidney in Response to Pregnancy
Source: Int J Mol Sci. 2022 Jun 3;23(11):6287. doi: 10.3390/ijms23116287 (PMC9181623; doi:10.3390/ijms23116287)
Supplement: Supplementary file 1 [file ijms-23-06287-s001.zip › ijms-1720592-supplemental materials/supplemental tables 1-2.pdf]

Scheme 1. List of primary antibodies used for western blotting analysis of protein abundance.

Primary Antibody	Dilution	Company and catalogue number
PI3 Kinase p85α	1/5,000	Millipore, 06-195
PI3 Kinase p110α	1/1,000	Cell Signaling Technology, 4249
Phospho-AKT (SerS473)	1/1,000	Cell Signaling Technology, 9271
Total-AKT	1/1,000	Cell Signaling Technology, 9272
Phospho-MAPK (Thr202/Tyr204)	1/1,000	Cell Signaling Technology, 4370
Total-MAPK	1/1,000	Cell Signaling Technology, 4695
Phospho-p38 MAPK (Thr180/Tyr182)	1/1,000	Cell Signaling Technology, 4511
Total-p38 MAPK	1/1,000	Cell Signaling Technology, 8690
Total JNK1	1/1,000	Cell Signaling Technology, 3708
Phospho-AMPKα (Thr172)	1/1,000	Cell Signaling Technology, 2531
Total-AMPKα	1/1,000	Cell Signaling Technology, 2532
PPAR-γ	1/200	Santa Cruz, sc-7273

Scheme 2. Primers used for qPCR analysis of gene expression. F: forward primer, R: reverse primer.

Gene name	Sequence (5' - 3')
Cdk1-F	ACTCGGCCTCTAAGCTCCT
Cdk1-R	AGGTTACGACGGACCCTCTC
Cdk2-F	CGGCTCGACACTGAGACTG
Cdk2-R	TTCTTGAGGTCCTGGTGCAG
Cdk4-F	CTTAGCCGAGCGTAAGGCTG
Cdk4-R	CCAGGCCGCTTAGAAACTGA
Cdc6-F	TCTGCAAGACTTCAAGAAGGAAG
Cdc6-R	AACACGATCATGGGGCCTTT
Cdc42-F	GGCGGAGAAGCTGAGGACA
Cdc42-R	ACCAACAGCACCATCACCAA
Ccne1-F	GACACAGCTTCGGGTCTGAG
Ccne1-R	CTGGAGCGGACTGAAAGGTC
Foxm1-F	CGGCCTGTGAGGGTCAAA
Foxm1-R	CTGATGTTTCACTCGGGGCA
Rb1-F	TGCATGGCTTTCAGATTCACC
Rb1-R	GCTGAGAGGACAAGCAGGTT
Nrf1-F	AGAAACGGAAACGGCCTCAT
Nrf1-R	CATCCAACGTGGCTCTGAGT
Nrf2-F	ATGGAGCAAGTTTGGCAGGA

Nrf-R	GCTGGGAACAGCGGTAGTAT
Tfam-F	TCCACAGAACAGCTACCCAA
Tfam-R	CCACAGGGCTGCAATTTTCC
Opa1-F	TGGGCTGCAGAGGATGGT
Opa-R	CCTGATGTCACGGTGTTGATG
Mfn1-F	TTGCCACAAGCTGTGTTCGG
Mfn1-R	TCTAGGGACCTGAAAGATGGGC
Mfn2-F	AGAGGCAGTTTGAGGAGTGC
Mfn2-R	ATGATGAGACGAACGGCCTC
Drp1-F	ATGCCAGCAAGTCCACAGAA
Drp1-R	TGTTCTCGGGCAGACAGTTT
Fis1-F	CAAAGAGGAACAGCGGGACT
Fis1-R	ACAGCCCTCGCACATACTTT
Hprt-F	CAGGCCAGACTTTGTTGGAT
Hprt-R	TTGCGCTCATCTTAGGCTTT
Ywhaz-F	AAACAGCTTTCGATGAAGCCA
Ywhaz-R	CATCTCCTTGGGTATCCGATGT
Polr2a-F	CACTGTCATCACCCCTGACC
Polr2a-R	ATACTGGCTGTTTCCCCTGC
